# Initial treatment efficacy and safety of durvalumab plus tremelimumab combination therapy in unresectable hepatocellular carcinoma in clinical practice

**DOI:** 10.1002/jgh3.70033

**Published:** 2024-10-04

**Authors:** Tetsu Tomonari, Joji Tani, Yasushi Sato, Hironori Tanaka, Akihiro Morishita, Koichi Okamoto, Yutaka Kawano, Masahiro Sogabe, Hiroshi Miyamoto, Tetsuji Takayama

**Affiliations:** ^1^ Department of Gastroenterology and Oncology, Institute of Biomedical Sciences Tokushima University Graduate School of Medicine Tokushima Japan; ^2^ Department of Gastroenterology and Neurology Kagawa University Graduate School of Medicine Kagawa Japan; ^3^ Department of Community Medicine for Gastroenterology and Oncology, Institute of Biomedical Sciences Tokushima University Graduate School of Medicine Tokushima Japan

**Keywords:** durvalumab, hepatocellular carcinoma, tremelimumab

## Abstract

**Background and Aims:**

We aimed to evaluate the efficacy and safety of durvalumab plus tremelimumab (Dur + Tre) combination therapy in patients with unresectable hepatocellular carcinoma (uHCC) in clinical practice.

**Methods:**

We retrospectively evaluated 37 patients with uHCC from our institutions between April 2023 and January 2024. Patients were divided into first‐ and later‐line groups for analysis of antitumor efficacy, adverse events (AEs), and transition rate to second‐line treatment according to the Response Evaluation Criteria in Solid Tumors (RECIST).

**Results:**

The disease control rate (DCR) for the first‐line group was 80.9%, which was significantly higher than that for the later‐line group (50%). The incidence of immune‐related AEs (irAEs) was 24.3%, with grade 3 or higher irAEs including increased transaminase (8.1%), diarrhea (8.1%), and adrenal insufficiency (2.7%). The rates of drug withdrawal and discontinuation owing to AEs were 23.8% and 19%, respectively, in the first‐line treatment and 31.2% and 12.5%, respectively, in the later‐line treatment, with no significant difference. Analysis of changes in liver reserve using the albumin–bilirubin (ALBI) score showed no obvious loss of liver reserve for up to 12 weeks. The transition rate from first‐ to second‐line therapy after progressive disease (PD) was as high as 94.7%.

**Conclusion:**

The efficacy and safety of Dur + Tre in clinical practice were comparable to those reported in a recent phase III trial. The first‐line Dur + Tre therapy had a higher DCR than that of the later lines, and the transition rate to second‐line therapy was considerably high, suggesting that Dur + Tre therapy would be more beneficial in first‐line treatment.

## Graphical abstract

In this study, we analyzed the changes in tumor markers, both AFP and DCP, which were linked to the tumor evaluation decision in RECIST, especially at the time of PD evaluation, with a significant increase in AFP and DCP after one month, suggesting that they may be useful in determining early efficacy.

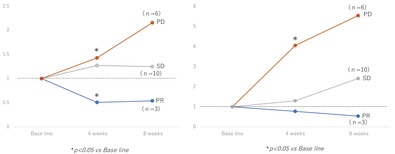

## Introduction

Hepatocellular carcinoma (HCC) is the fifth leading cause of cancer death worldwide, necessitating urgent development of cure.^1^, ^2^ In recent years, drug therapy for advanced HCC has made remarkable progress, and a variety of drugs have been approved.

The phase III IMbrave150 trial, which reported the efficacy of atezolizumab (a programmed cell death ligand 1 [PD‐L1]‐targeting monoclonal antibody) plus bevacizumab (a vascular endothelial growth factor [VEGF]‐A‐targeting monoclonal antibody) (Atezo+Bev) as a first‐line drug therapy, showed a significant overall survival (OS) benefit compared with sorafenib (hazard ratio [HR], 0.58; 95% confidence interval [CI], 0.42–0.79).^3^ Furthermore, a new immune checkpoint‐based drug therapy, durvalumab (a PD‐L1‐targeting monoclonal antibody) plus tremelimumab (a cytotoxic T‐lymphocyte‐associated antigen‐4‐blocking antibody) (Dur + Tre), has been reported to be effective in the treatment of unresectable HCC (uHCC), based on the results of the phase III HIMALAYA trial (HR, 0.78; 95% CI, 0.65–0.93), which also demonstrated a significant survival benefit when compared with sorafenib.^4^ Although the results of this trial have allowed Dur + Tre to be used as first‐line therapy for HCC along with Atezo+Bev, its outcome in actual clinical practice,^5^, ^6^ including efficacy and safety, especially when used in the second line and beyond, remains to be clarified. This study aimed to analyze the efficacy and safety of Dur + Tre in clinical practice by retrospectively collecting Dur + Tre cases, including later‐line cases.

## Methods

### Patient selection and diagnosis of HCC

This study retrospectively evaluated the efficacy and safety of Dur + Tre (AstraZeneca plc, Cambridge, UK) therapy for uHCC initiated at Tokushima University Hospital and Kagawa University Hospital from April 2023 to January 2024.

Case selection criteria were based on the phase III HIMALAYA study.^4^ Eligible patients had nodules evaluable by the Response Evaluation Criteria in Solid Tumors (RECIST)^7^ or modified RECIST (mRECIST),^8^ a Child–Pugh (CP) class A (CP‐A), an Eastern Cooperative Oncology Group Performance Status (ECOG‐PS) score of 0 or 1,^9^ and a Barcelona Clinic Liver Cancer (BCLC) stage of B or C.^10^ Although some of the cases included CP‐B, they were dosed according to the criteria of the GO30140 study,^11^ which included anti‐PD‐L1 for uHCC. In addition, Dur + Tre was preferentially initiated as the first‐line treatment in patients with concerns about bleeding or proteinuria caused by VEGF inhibitors.

The diagnosis of HCC was made according to the Liver Cancer Study Group guidelines in Japan and by pathology or imaging evaluation.^5^ Typically, contrast‐enhanced computed tomography or magnetic resonance imaging that showed tumors that were dark in the early phase and washed out in the late phase were diagnosed as HCC.

Consistent with the criteria of previous clinical trials, the treatment of choice for progressive disease (PD) on radiologic imaging after Dur + Tre therapy was pharmacotherapy when hepatic reserve function at the time of PD was CP‐A and the performance status (PS) was 0.^12^, ^13^, ^14^ In case the patient failed to meet any of these criteria, options such as transarterial embolization (TAE) or transarterial chemoembolization (TACE), as well as hepatic arterial infusion chemotherapy (HAIC), were used. Additionally, best supportive care (BSC) was conducted for patients deemed unsuitable for TAE, TACE, or HAIC, or for those whose hepatic reserve function had declined to CP‐C.

This study was conducted in accordance with the guidelines of the Helsinki Declaration of 1975. The protocol of this study was approved by the Institutional Review Board of Tokushima University Hospital (number: 3816) and the participating facilities.

### Treatment protocol

The Dur + Tre regimen entails administering 300‐mg tremelimumab and 1500‐mg durvalumab as the initial dose, with subsequent administrations of 1500 mg durvalumab every 4 weeks following the second dose. In case of a serious adverse event (AE), such as an unfavorable grade 2 or 3 AE, Dur + Tre therapy was discontinued until the patient's condition improved to a lower grade than when the initial AE occurred. Resumption of therapy was considered when the patient's condition improved to grade 1 or lower.

### Patient outcomes and assessment

Patients who received a minimum of 4 weeks of Dur + Tre therapy were included in the present analysis. Safety parameters were scrutinized by laboratory analysis of blood and urine samples for hematological and biochemical indices and by physical assessment. AEs attributed to medication were evaluated utilizing the Common Terminology Criteria for AEs, version 5.0. AEs were determined by the outpatient attending physician.

Response to Dur + Tre therapy was assessed at intervals of 8 weeks using the RECIST and mRECIST criteria. The overall response rate (ORR) was delineated as the sum of complete response (CR) and partial response (PR), while the disease control rate (DCR) encompassed CR, PR, or stable disease (SD). In this analysis, the treatment response reported in the Results section represents the best observed response. Progression‐free survival (PFS) was defined as the duration from the initiation of treatment with Dur + Tre to either radiological progression or death from any cause.

The assessment of hepatic functional reserve involved the utilization of CP scoring alongside modified albumin–bilirubin (ALBI) (mALBI) grading. The determination of the mALBI grade was based on the assessment of total bilirubin and serum albumin levels. Patients who ceased Dur + Tre treatment within 4 weeks were excluded from the analysis pertaining to the alteration in the ALBI score.

### Statistical analysis

Binomial variables underwent evaluation utilizing Fisher's exact test, while continuous variables were assessed through the Mann–Whitney *U* test. Changes in tumor markers and were similarly tested using the Mann–Whitney *U* test. ALBI scores were analyzed using the Wilcoxon signed‐rank test. Statistical significance was established at a *P* value of <0.05. PFS was analyzed employing the Kaplan–Meier method alongside the log‐rank test. All statistical computations were performed using Easy R, version 1 (Saitama Medical Center, Jichi Medical University, Saitama, Japan).^15^

## Results

### Patient characteristics

Three patients were excluded owing to incomplete initial radiological assessments, resulting in 37 patients being included in the study (Table 1). The median age of the patients was 73 years (interquartile range, 67–77), of which eight (21.6%) were female and 32 (86.5%) exhibited an ECOG‐PS of 0. Among all patients, six (16.2%) tested positive for hepatitis B virus antigen, 13 (35.1%) tested positive for hepatitis C virus antibody, and the pretreatment CP score ranged from 5 to 8.

**Table 1:** Characteristics of patients with unresectable advanced hepatocellular carcinoma (HCC) treated with durvalumab plus tremelimumab therapy

Characteristics	All (*n* = 37)	First line (*n* = 21)	Later line (*n* = 16)	*P* value
Age, median [Quartiles], (years)	73 [67–77]	76 [72–78]	72 [62–73]	0.07
Sex (male/female), *n*	29/8	16/5	13/3	1
ECOG‐PS (0/1), *n*	32/5	18/3	14/2	1
Etiology (HBV/HCV/NBNC), *n*	6/13/18	0/9/12	6/15/7	0.33
Platelets, median [Quartiles], (10^4^/μL)	15.3 [9.0–21.4]	16.4 [13.5–19.4]	14.8 [8.8–17.5]	0.50
Child–Pugh score (5/6/7/8), *n*	15/18/2/1	6/12/2/1	9/6/0/0	0.09
mALBI Grade (1/2a/2b/3), *n*	6/10/21/0	3/5/13/0	3/5/8/0	0.56
Portal vein invasion (absent/present), *n*	32/5	21/0	11/5	0.01
Extrahepatic spread (absent/present), *n*	28/9	16/5	12/4	1
AFP, median [Quartiles] (ng/ml)	41 [5–1124]	18 [4–353]	337 [10–4214]	0.95
BCLC stage (A/B/C), *n*	1/22/14	1/14/6	0/8/8	0.31
Treatment line (first line/second line/third line/fourth line fifth line/sixth line), *n*	21/7/4/0/2/3	21/0/0/0/0/0	0/7/4/0/2/3	0.49
Previous treatment history (Atezo+Bev/Lenvatinib)	14/9	0/0	14/9	‐

AFP, alpha‐fetoprotein; Atezo+Bev, Atezolizumab+Bevacizumab; BCLC, Barcelona Clinic Liver Cancer; ECOG‐PS, Eastern Cooperative Oncology Group performance status; HBV, hepatitis B virus; HCV, hepatitis C virus; mALBI, modified albumin–bilirubin; NBNC, non‐B non C.

The mALBI grades at the start of Dur + Tre therapy ranged from 1 to 2a, including mALBI one in six patients, 2a in 10 patients, and 2b in 21 patients. The median alpha‐fetoprotein (AFP) level was 41 ng/mL (interquartile range, 5–1124 ng/mL). In addition, 23 patients had stage B BCLC and 14 patients had stage C BCLC. Among the cases of portal vein invasion, there was one case of Vp1, one case of Vp2, three cases of Vp3, and no cases of Vp4. Of these patients, 21 received Dur + Tre as initial therapy, and the subsequent treatment lines were second line in seven, third line in four, fifth line in two, and sixth line in three patients. The breakdown of later lines included 14 cases of Atezo+Bev (87.5%), including nine cases of lenvatinib (56.3%) and five cases of sorafenib (31.3%), and two cases of ramucirumab (12.5%) and two of regorafenib (12.5%) out of 16 cases. Comparing the patient backgrounds of the first‐ and later‐line treatments, portal vein invasion was significantly more common in the later‐line treatment group (*P* < 0.05).

### Treatment effect

The median duration of observation in this study was 195 days (interquartile range 143–370). The median number of Dur + Tre courses was three (interquartile range 2–4). The treatment response outcomes in accordance with the RECIST (version 1.1) and mRECIST are outlined in Table 2. In all patients, the ORR was 16.2%, with a CR rate of 2.7% and PR rate of 13.5%. SD and PD were noted in 54.1% and 32.4% of the patients, respectively, resulting in a DCR of 67.6%. In first‐line treatment, the ORR was 23.8%, compared with 6.3% in subsequent treatments, with no significant difference. The DCR was significantly higher in the first‐line treatment (83.9%) than in subsequent treatments (50%) (*P* < 0.05).

**Table 2:** Response to treatment with durvalumab plus tremelimumab for hepatocellular carcinoma (HCC)

Evaluation	RECIST, ver. 1.1			mRECIST		
	All, *n* (%) (*n* = 37)	First line, *n* (%) (*n* = 21)	Later line, *n* (%) (*n* = 16)	All, *n* (%) (*n* = 37)	First line, *n* (%) (*n* = 21)	Later line, *n* (%) (*n* = 16)
Complete response	1 (2.7)	1 (4.8)	0 (0)	1 (2.7)	1 (4.8)	0 (0)
Partial response	5 (13.5)	4 (19.0)	1 (6.3)	5 (13.5)	4 (19.0)	1 (6.3)
Stable disease	19 (51.4)	12 (57.1)	7 (43.7)	20 (54.1)	13 (61.9)	7 (43.7)
Progressive disease	12 (32.4)	4 (19.0)	8 (50.0)	11 (29.7)	3 (14.3)	8 (50.0)
Objective response rate (%)	16.2	23.8	6.3	16.2	23.8	6.3
Disease control rate (%)	67.6	83.9*	50.0	70.3	85.7*	50.0

* *P* < 0.05 versus later line.

mRECIST, modified Response Evaluation Criteria in Solid Tumors; RECIST, Response Evaluation Criteria in Solid Tumors.

Similar results were obtained according to the mRECIST. In all patients, CR and PR were observed in 2.7% and 13.5% of the patients, respectively, resulting in an ORR of 16.2%. SD and PD were noted in 54.1% and 29.7% of the patients, respectively, resulting in a DCR of 70.3%. The DCR was significantly higher in the first‐line treatment (85.7%) than in subsequent treatments (50%) (*P* < 0.05).

**Figure 1:**
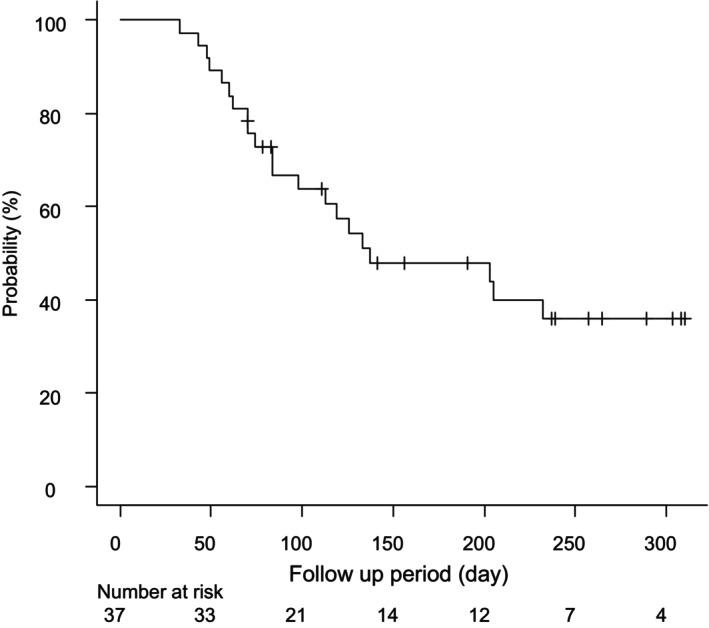
The progression‐free survival of all 37 patients treated with durvalumab + tremelimumab analyzed using a Kaplan–Meier curve.

The median PFS, as determined by the RECIST, was 4.5 months (Fig. 1). No statistically significant disparity in median PFS was observed between the first‐line cohort (6.5 months, 95% CI 2.4 to not available [NA] months) and the subsequent‐line cohort (3.6 months, 95% CI 1.9 to NA months) (Fig. S1) (*P* = 0.31). Furthermore, the median OS was not reached.

### Changes in AFP and des‐γ‐carboxy prothrombin (DCP) levels

**Figure 2:**
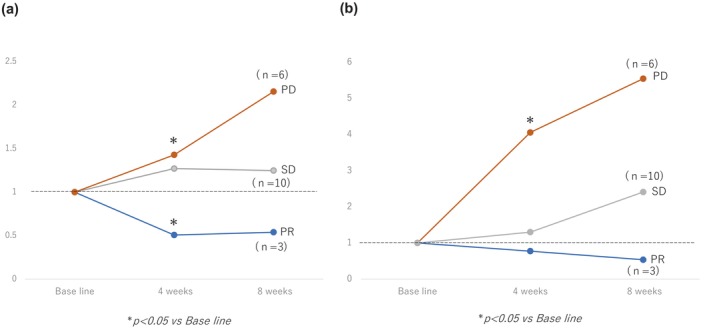
(a) The transition of the AFP value after the administration of treatment with durvalumab + tremelimumab at baseline and 4, 8, and 12 weeks. (b) The transition of the DCP value after the administration of treatment with durvalumab + tremelimumab at baseline and 4, 8, and 12 weeks.

Figure 2 shows the changes in AFP and DCP values for up to 12 weeks of Dur + Tre treatment. The patients who were evaluated for PR by the RECIST showed that the AFP ratio at 4 and 8 weeks significantly decreased and significantly increased, respectively, in patients who were evaluated for imaging PD. Similarly, the DCP ratio at 4 and 8 weeks was significantly decreased in patients with PR and significantly increased at 4 and 8 weeks in patients with PD.

### Secondary treatment and transition rate following the progression of dur + Tre treatment

During the observation period, radiological confirmation of PD was evident in 19 of 37 patients who underwent Dur + Tre treatment (Table 3). In instances where PD was identified through imaging, the transition to systemic therapy (multimolecular target agent [MTAs] and combined immunotherapy) was considered, particularly for patients exhibiting CP‐A and PS‐0 or 1. Within this cohort, subsequent therapeutic interventions were pursued by 18 patients (94.7%), including various modalities, such as drug therapy (Atezo+Bev, *n* = 1; lenvatinib, *n* = 7; cabozantinib, *n* = 2; ramucirumab, *n* = 2; and sorafenib, *n* = 1; 68.4%), TAE or TACE (*n* = 3; 15.7%), HAIC (*n* = 2; 10.5%), and, ultimately, BSC (*n* = 1; 5.3%). There was no significant difference in the transition rate from the first and later lines to posttreatment, either by drug therapy or catheterization (TAE/TACE or HAIC).

**Table 3:** Secondary treatment after imaging progression on durvalumab plus tremelimumab therapy

Characteristics	All, *N* (%) (*n* = 19)	First line, *N* (%) (*n* = 12)	Later line, *N* (%) (*n* = 7)	*P* value
Post‐therapy (other than BSC)	18 (94.7)	11 (91.7)	7 (100)	1
Systemic therapy	13 (68.4)	7 (63.6)	6 (85.7)	0.33
TAE/TACE	3 (15.7)	2 (18.2)	1 (14.3)	1
HAIC	2 (10.5)	2 (18.2)	0 (0)	1
BSC	1 (5.3)	1 (9.1)	0 (0)	1

BSC, best supportive care; HAIC, hepatic arterial infusion chemotherapy; TAE/TACE, transcatheter embolization/chemoembolization.

### Adverse events

AEs observed during the administration of Dur + Tre are listed in Table 4. The most prevalent AE was pruritus (all grades, *n* = 9/37 [24.3%] and grade 3, *n* = 0/36 [0%]), followed by fatigue (all grades, *n* = 6/37 [16.2%] and grade 3, *n* = 0 [0%]), increased transaminase (all grades, *n* = 5/37 [13.5%] and grade 3, *n* = 3 [8.1%]), diarrhea (all grades, *n* = 5/37 [13.5%] and grade 3, *n* = 3 [8.1%]), appetite loss (all grades, *n* = 4/37 [10.8%] and grade 3, *n* = 0 [0%]), fever (all grades, *n* = 3/37 [8.1%] and grade 3, *n* = 0 [0%]), hypothyroidism (all grades, *n* = 2/37 [5.4%] and grade 3, *n* = 0 [0%]), and adrenal insufficiency (all grades, *n* = 2/37 [5.4%] and grade 3, *n* = 1 [2.7%]). Four of the six cases of fatigue and all four cases of appetite loss were AEs associated with adrenal insufficiency and hypothyroidism. Furthermore, one case involved combined grades 3 or 4 AEs: liver injury (grade 3) and acute renal failure (grade 4) [2.7%]. No deaths resulted from AEs.

**Table 4:** Adverse events associated with durvalumab plus tremelimumab treatment

Event	All (*n* = 37)		First line (*n* = 21)		Later line (*n* = 16)		*P* value	
	Any Grade	Grade 3	Any Grade	Grade 3	Any Grade	Grade 3	Any Grade	Grade 3
Pruritus	9 (24.3)	0 (0)	6 (28.6)	0 (0)	3 (18.8)	0 (0)	0.70	—
Fatigue	6 (16.2)	0 (0)	2 (9.5)	0 (0)	4 (25.0)	0 (0)	0.37	—
Increased transaminase	5 (13.5)	3 (8.1)	3 (14.3)	2 (9.5)	2 (12.5)	1 (6.3)	1	1
Diarrhea	5 (13.5)	3 (8.1)	4 (19.0)	2 (9.5)	1 (6.3)	1 (6.3)	0.36	1
Appetite loss	4 (10.8)	0 (0)	1 (4.8)	0 (0)	3 (18.8)	0 (0)	0.30	—
Fever	3 (8.1)	0 (0)	2 (9.5)	0 (0)	1 (6.3)	0 (0)	1	—
Hypothyroidism	2 (5.4)	2 (5.4)	2 (9.5)	2 (9.5)	0 (0)	0 (0)	0.50	0.50
Adrenal insufficiency	2 (5.4)	1 (2.7)	0 (0)	0 (0)	2 (12.5)	1 (6.3)	0.18	0.43
Interstitial pneumonia	1 (2.7)	0 (0)	1 (4.8)	0 (0)	0 (0)	0 (0)	1	—
Hyperthyroidism	1 (2.7)	0 (0)	1 (4.8)	0 (0)	0 (0)	0 ()	1	—
Myositis	1 (2.7)	1 (2.7)	0 (0)	0 (0)	1 (6.3)	1 (6.3)	1	1
Acute kidney injury	1 (2.7)	1 (0)	1 (4.8)	1 (4.8)	0 (0)	0 (0)	1	1
Decreased platelet count	1 (2.7)	1 (2.7)	0 (0)	0 (0)	1 (6.3)	1 (6.3)	1	1
Withdrawal	10 (27)		5 (23.8)		5 (31.2)		0.72	
Discontinuation	6 (16.2)		4 (19.0)		2 (12.5)		0.68	

Among these instances, steroid therapy was necessitated in nine cases (24.3%), with high‐dose steroids (≥40 mg) employed in seven cases (18.9%). Infliximab was also introduced in two cases (5.4%) of enteritis refractory to high‐dose steroids, and encouragement of induction resulted in symptomatic improvement. When these cases were analyzed by dividing them into first and later lines, medication withdrawal was 27% overall, with 23.8% in the first line and 31.2% in the later line, which was not significantly different (*P* = 0.72). Discontinuation was 16.2% overall, with 19% in the first‐line and 12.5% in the later‐line treatment, which was also not significantly different (*P* = 0.68).

### Effect of dur + Tre on the ALBI score over the treatment period

**Figure 3:**
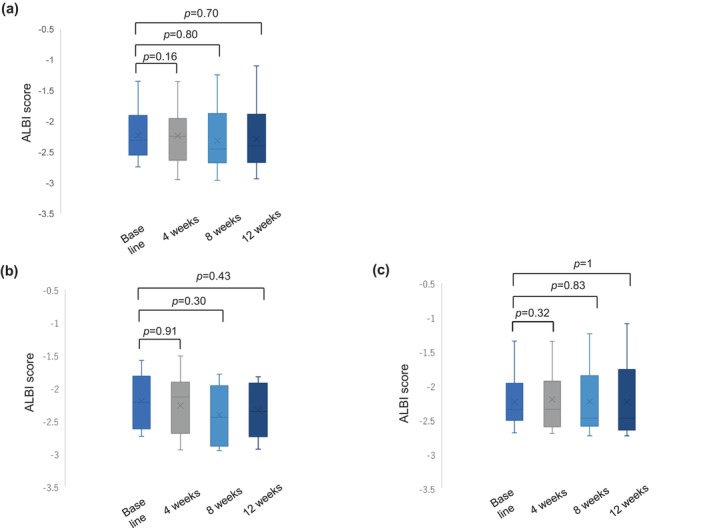
(a) The transition of hepatic functional reserve after the administration of treatment with durvalumab + tremelimumab at baseline and 4, 8, and 12 weeks. (b) The transition of hepatic functional reserve in first‐line treatment after the introduction of treatment with durvalumab + tremelimumab at baseline and 4, 8, and 12 weeks. (c) The transition of hepatic functional reserve in later‐line treatment after the introduction of treatment with durvalumab + tremelimumab at baseline and 4, 8, and 12 weeks.

Following the commencement of Dur + Tre treatment, alterations in ALBI scores over a period of up to 12 weeks were assessed in a cohort of 20 patients (Fig. 3a). The median ALBI scores at baseline and weeks 4, 8, and 12 were documented as −2.31 (with quartiles ranging from −1.95 to −2.52), −2.24 (with quartiles ranging from −1.98 to −2.61), −2.44 (with quartiles ranging from −1.98 to −2.62), and − 2.39 (with quartiles ranging from −1.97 to −2.65), respectively. Notably, there were no statistically significant disparities in ALBI scores compared with baseline at weeks 4, 8, and 12, as depicted in Figure 3. Upon stratification of these patients into two cohorts, namely, the first‐ (Fig. 3b) and second‐line groups (Fig. 3c), no significant differences were observed in the ALBI scores from baseline to week 12 in either group.

## Discussion

To date, few studies have assessed the real‐world clinical effectiveness and safety profile of Dur + Tre for uHCC, and few have documented the rates of transition to second‐line treatment. In the present investigation, we observed that the implementation of Dur + Tre therapy in the real world exhibited an outcome comparable to that observed in the HIMALAYA trial in terms of clinical effectiveness and safety profile.^4^ The real‐world response rate and DCR in this study were 23.8% and 83.9%, respectively, which were comparable to the STRIDE regimen in the HIMALAYA trial, which had a response rate of 20.1% and DCR of 60.1%.^4^

Regarding AEs, caution is required with regard to immune‐related AEs (irAEs) because drug therapy uses two immune checkpoint inhibitors; the data from the HIMALAYA study showed that the incidence of irAEs was 35.8% and the use of high‐dose steroids was 20.1%.^4^ In the present study, the incidence of irAEs was 24.3%, and the use of high‐dose steroids was 16.2%, which was similar to the results of the HIMALAYA study.

In contrast, the rate of discontinuation owing to AEs was slightly higher in the present study (16.2%), this may be due to the inclusion of cases after the later‐line therapy and of CP‐B cases.

Dur + Tre therapy has been associated with a high incidence of irAE enteritis.^4^

The American Society of Clinical Oncology guidelines recommend starting with methylprednisolone at 1–2 mg/kg/day for grades 3–4 irAE enteritis. In case no response is observed after 2–5 days (grade 3, 3–5 days and grade 4, 2–3 days), infliximab should be initiated.^16^ In this study, we started treatment with high‐dose steroids for grade 3 or higher irAE enteritis; however, the patients were unresponsive to steroids. Thus, we switched to infliximab, which was successful in two cases. This highlights the importance of recognizing that Dur + Tre therapy can result in steroid‐refractory irAE enteritis, necessitating infliximab induction.

Previously, Atezo+Bev was the first‐line therapy for uHCC based on the phase III IMbrave150 trial results.^3^ However, the phase III HIMALAYA trial results introduced Dur + Tre as an alternative,^5^ leading to debate over their use for uHCC. The American Association for the Study of Liver Diseases guidelines suggest using Dur + Tre for patients with no bleeding risk, as Atezo+Bev has an associated bleeding risk due to bevacizumab.^6^ Thus, Dur + Tre is recommended as a first‐line option for patients at risk of bleeding.

When comparing the first‐line and subsequent treatment outcomes of Dur + Tre therapy, it became clear that the first‐line group showed a significantly superior DCR (83.9%) to that of the later‐line groups (50%), where 87.5% of cases were initially treated with Atezo+Bev therapy. Shimose et al. reported a better DCR with Dur + Tre therapy in the first line than in the later line, suggesting superior disease control in the first‐line setting.^17^ Considering the reported moderate correlation between DCR and OS in immunotherapy alone for HCC,^18^, ^19^ first‐line Dur + Tre therapy may yield a more favorable outcomes compared with later‐line treatment.

Compared with the initial results of Atezo+Bev in actual clinical practice, Ando et al. reported no difference in ORR between the first and later lines.^20^ However, in the present clinical data, Dur + Tre demonstrates significantly better disease control in the first line than in the later line, suggesting that Dur + Tre may be more effective when administered before Atezo+Bev. However, no studies have directly compared Atezo+Bev and Dur + Tre as a first‐line treatment option. Therefore, further research on this matter is warranted.

Furthermore, we found that the overall transition rate to later‐line therapy was 94.7%, with a 68.4% transition rate to systemic therapy, whereas previous reports of Atezo+Bev for uHCC showed an overall transition rate to later‐line therapy of 68.3% and a 46.3% transition rate to systemic therapy. This indicates that Dur + Tre therapy could have a higher transition rate to later‐line therapy than Atezo+Bev therapy^17^ and may be effective as part of sequential therapy aimed at prolonging survival.

Based on these reports and the results of our study, we suggest that Dur + Tre therapy is preferable as a first‐line setting.

Regarding tumor markers, DCP may not be useful as a tumor marker at it is generated from hypoxic HCC due to the VEGF inhibitors included in previous HCC systemic therapies.^21^ However, Dur + Tre therapy is pure immunotherapy that does not include VEGF inhibitors, suggesting that it directly reflects the antitumor effect. In the present study, a significant increase was observed in both AFP and DCP in the PD group at 4 weeks, indicating their potential as early response markers for immunotherapy against HCC. This suggests the possibility of identifying patients who may benefit from an early switch in treatment.

Regarding hepatic reserve function, the results of the HIMALAYA study reported that the Dur + Tre combination did not decrease hepatic reserve during dosing,^4^ and real‐world clinical data showed similar results, with no significant decrease in the ALBI score from baseline to 12 weeks in the analysis of liver reserve using the ALBI score. The ALBI score did not decrease significantly from baseline to 12 weeks. These results were similar in both the first and later lines, suggesting that the drug is effective as one that is less likely to reduce hepatic reserve capacity in sequential drug therapy for HCC.

This study had several limitations, including the short observation period, retrospective study design, and small sample size. Therefore, a large prospective study is needed to further analyze our findings.

Our data suggested that Dur + Tre treatment has a favorable disease control effect in first‐line therapy and does not deteriorate liver reserve; therefore, the rate of transition to subsequent MTA treatment is reasonably high. Therefore, this study suggests that it is desirable to use Dur + Tre therapy as the first‐line treatment strategy for HCC, which may contribute to prolonging the prognosis of drug therapy for HCC.

## Ethics approval statement

This study was conducted in accordance with the guidelines of the Helsinki Declaration of 1975. The protocol of this study was approved by the Institutional Review Board of Tokushima University Hospital (number: 3816) and the participating facilities.

## Patient consent statement

Informed consent was obtained in the form of opt‐out on the website. Those who rejected were excluded.

## Supporting information


**Figure S1.** The progression‐free survival of first‐line and later ‐line patients treated with durvalumab + tremelimumab analyzed using a Kaplan–Meier curve.

## Data Availability

The data that support the findings of this study are available from the corresponding author upon reasonable request.

## References

[1] Forner A , Reig M , Bruix J . Hepatocellular carcinoma. Lancet. 2018; 391: 1301–1314. 29307467 10.1016/S0140-6736(18)30010-2

[2] Nault JC , Galle PR , Marquardt JU . The role of molecular enrichment on future therapies in hepatocellular carcinoma. J. Hepatol. 2018; 69: 237–247. 29505843 10.1016/j.jhep.2018.02.016

[3] Finn RS , Qin S , Ikeda M *et al*. Atezolizumab plus Bevacizumab in Unresectable Hepatocellular Carcinoma. N. Engl. J. Med. 2020; 382: 1894–1905. 32402160 10.1056/NEJMoa1915745

[4] Abou‐Alfa GK , Lau G , Kudo M *et al*. Tremelimumab plus durvalumab in unresectable hepatocellular carcinoma. NEJM Evid. 2022; 1: EVIDoa2100070. 38319892 10.1056/EVIDoa2100070

[5] Hasegawa K , Takemura N , Yamashita T *et al*. Clinical Practice Guidelines for Hepatocellular Carcinoma: The Japan Society of Hepatology 2021 version (5th JSH‐HCC Guidelines). Hepatol. Res. 2023; 53: 383–390. 36826411 10.1111/hepr.13892

[6] Singal AG , Llovet JM , Yarchoan M *et al*. AASLD Practice Guidance on prevention, diagnosis, and treatment of hepatocellular carcinoma. Hepatology (Baltimore, MD). 2023; 78: 1922–1965. 10.1097/HEP.0000000000000466 PMC10663390 37199193

[7] Schwartz LH , Seymour L , Litière S *et al*. RECIST 1.1 ‐ Standardisation and disease‐specific adaptations: Perspectives from the RECIST Working Group. Eur. J. Cancer. 2016; 62: 138–145. 27237360 10.1016/j.ejca.2016.03.082 PMC5737786

[8] Lencioni R , Llovet JM . Modified RECIST (mRECIST) assessment for hepatocellular carcinoma. Semin. Liver Dis. 2010; 30: 52–60. 20175033 10.1055/s-0030-1247132 PMC12268942

[9] Oken MM , Creech RH , Tormey DC *et al*. Toxicity and response criteria of the Eastern Cooperative Oncology Group. Am. J. Clin. Oncol. 1982; 5: 649–655. 7165009

[10] Llovet JM , Villanueva A , Marrero JA *et al*. Trial design and endpoints in hepatocellular carcinoma: AASLD consensus conference. Hepatology (Baltimore, MD). 2021; 73: 158–191. 10.1002/hep.31327 32430997

[11] Lee MS , Ryoo BY , Hsu CH *et al*. Atezolizumab with or without bevacizumab in unresectable hepatocellular carcinoma (GO30140): an open‐label, multicentre, phase 1b study. Lancet Oncol. 2020; 21: 808–820. 32502443 10.1016/S1470-2045(20)30156-X

[12] Bruix J , Qin S , Merle P *et al*. Regorafenib for patients with hepatocellular carcinoma who progressed on sorafenib treatment (RESORCE): a randomised, double‐blind, placebo‐controlled, phase 3 trial. Lancet. 2017; 389: 56–66. 27932229 10.1016/S0140-6736(16)32453-9

[13] Zhu AX , Kang YK , Yen CJ *et al*. Ramucirumab after sorafenib in patients with advanced hepatocellular carcinoma and increased alpha‐fetoprotein concentrations (REACH‐2): a randomised, double‐blind, placebo‐controlled, phase 3 trial. Lancet Oncol. 2019; 20: 282–296. 30665869 10.1016/S1470-2045(18)30937-9

[14] Abou‐Alfa GK , Meyer T , Cheng AL *et al*. Cabozantinib in Patients with Advanced and Progressing Hepatocellular Carcinoma. N. Engl. J. Med. 2018; 379: 54–63. 29972759 10.1056/NEJMoa1717002 PMC7523244

[15] Kanda Y . Investigation of the freely available easy‐to‐use software 'EZR' for medical statistics. Bone Marrow Transplant. 2013; 48: 452–458. 23208313 10.1038/bmt.2012.244 PMC3590441

[16] Schnipper LE , Davidson NE, Wollins DS *et al*. American Society of clinical oncology statement: a conceptual framework to assess the value of cancer treatment options. J. Clin. Oncol. 2015; 33: 2563–2577. 26101248 10.1200/JCO.2015.61.6706 PMC5015427

[17] Shimose S , Saeki I , Tomonari T *et al*. Initial clinical experience with durvalumab plus tremelimumab in patients with unresectable hepatocellular carcinoma in real‐world practice. Oncol. Lett. 2024; 28: 397. 38979550 10.3892/ol.2024.14530 PMC11228928

[18] Xu H , Cao D , Zheng Y *et al*. Potential predictors for survival in hepatocellular carcinoma patients treated with immune checkpoint inhibitors: a meta‐analysis. Int. Immunopharmacol. 2021; 100: 108135. 34530205 10.1016/j.intimp.2021.108135

[19] Sangro B , Chan SL , Kelley RK *et al*. Four‐year overall survival update from the phase III HIMALAYA study of tremelimumab plus durvalumab in unresectable hepatocellular carcinoma. Ann. Oncol. 2024; 35: 448–457. 38382875 10.1016/j.annonc.2024.02.005

[20] Ando Y , Kawaoka T , Kosaka M *et al*. Early Tumor Response and Safety of Atezolizumab Plus Bevacizumab for Patients with Unresectable Hepatocellular Carcinoma in Real‐World Practice. Cancers (Basel). 2021; 13: 3958. 34439111 10.3390/cancers13163958 PMC8394131

[21] Murata K , Suzuki H , Okano H , Oyamada T , Yasuda Y , Sakamoto A . Hypoxia‐induced des‐gamma‐carboxy prothrombin production in hepatocellular carcinoma. Int. J. Oncol. 2010; 36: 161–170. 19956845

