# Supplementary material for: Initial treatment efficacy and safety of durvalumab plus tremelimumab combination therapy in unresectable hepatocellular carcinoma in clinical practice
Source: JGH Open. 2024 Oct 4;8(10):e70033. doi: 10.1002/jgh3.70033 (PMC11450737; doi:10.1002/jgh3.70033)
Supplement: Supplementary file 1 — Figure S1. The progression‐free survival of first‐line and later ‐line patients treated with durvalumab + tremelimumab analyzed using a Kaplan–Meier curve. [file JGH3-8-e70033-s001.pptx]

## Slide 1
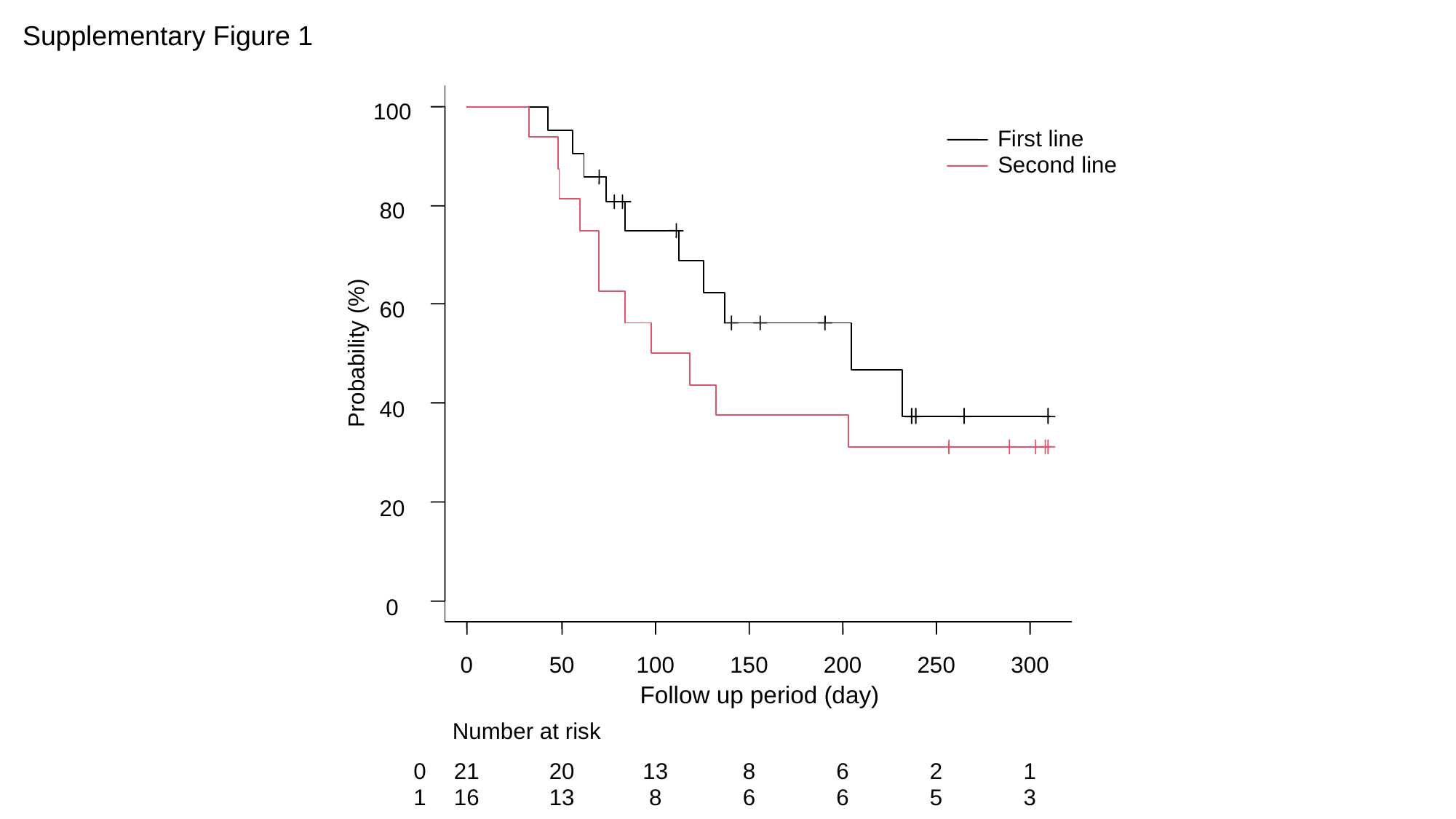

Supplementary Figure 1
100
First line
Second line
 80
 60
Probability (%)
 40
 20
 0
0
50
100
150
200
250
300
Number at risk
0
21
20
13
8
6
2
1
1
16
13
8
6
6
5
3
Follow up period (day)
